# Advancing global health equity: the transformative potential of community-based surveillance in developing countries

**DOI:** 10.3389/fpubh.2023.1294686

**Published:** 2023-12-07

**Authors:** Juan S. Izquierdo Condoy, Andrea Tello-De-la-Torre, Patricio Espinosa Del Pozo, Esteban Ortiz-Prado

**Affiliations:** ^1^One Health Research Group, Universidad de Las Américas, Quito, Ecuador; ^2^Facultad de Ciencias Médicas, Universidad Central del Ecuador, Quito, Ecuador

**Keywords:** community based surveillance, global health, community health workers, low income, Ecuador

## Introduction

In community settings, medical surveillance is an effective strategy for infectious disease control. It involves systematic epidemiological surveillance, facilitating the collection, analysis, and dissemination of health data. This process is vital for mitigating outbreak impacts and managing disease spread. The development and administration of public health programs within these frameworks are key to enhancing preparedness for future outbreaks, a necessity in the context of continual health threats (1).

Although surveillance strategies have classically relied predominantly on data from institutional sources like hospitals and public records, the reach of such methods has been shown to be limited, particularly in low- and middle-income countries. Such limitations stem from factors like inadequate healthcare infrastructure, cultural practices in rural areas, and incomplete civil registration systems (2–4).

Addressing these challenges, community-based surveillance, a well-established and historically significant tool in health systems, has seen a broadening of its scope. Initially focused on infectious diseases, this method of surveillance has expanded its applicability to encompass a diverse range of healthcare contexts (5–9). Reflecting on its conceptual evolution over decades, as detailed by Rojanaworarit (10), community-based surveillance now plays a crucial role in monitoring and responding to various health events, from disease outbreaks to environmental hazards, thereby shaping public health actions and policies. Notably, during health crises like pandemics, community-based systems have been found to be remarkably effective, sometimes surpassing formal healthcare systems in their efficacy (9).

## The contemporary relevance of community surveillance

Even today, developing nations face unique challenges in relation to health services, including geographic inaccessibility for populations residing in remote areas, and difficulty in accessing health services resulting in high health burdens. The incorporation of community-based surveillance stands as a prospective solution to bridge these gaps (4). Despite global efforts like those by the WHO, further concentration on establishing specialized frameworks for community-based surveillance systems remains imperative, especially for developing nations (11, 12).

## The call for structured guidelines and control frameworks

In light of the increasing urgency to respond rapidly to disease outbreaks and the limitations of conventional surveillance systems, we argue for the establishment of comprehensive guidance and regulatory frameworks for community surveillance systems. These should aim specifically at fortifying community surveillance systems, particularly in settings that are under-resourced and prone to crises. Drawing upon evidence from a systematic review by McGowan et al. (13), we advocate for the development of these rigorous frameworks, underlining the fundamental importance of community-based surveillance in the context of early detection and rapid response. This approach not only aligns with the International Health Regulations but also addresses critical gaps in surveillance mechanisms, especially in resource-limited settings (13).

## Effectiveness and sustainability

Aligned with the principles of participatory community engagement, effective community surveillance systems gain strength from their social context (14). The smaller geographical size of these communities fosters a more intimate and interconnected environment, which is crucial for rapid disease reporting and tracking. Close-knit relationships among community members enhance trust and communication, leading to more active local involvement and prompt sharing of health-related information (13). This familiarity not only facilitates quality assurance and technological adoption for real-time decision-making but also ensures a higher degree of responsiveness and accuracy in surveillance activities. Studies have shown that such social dynamics play a vital role in the success of community-based health initiatives (15). The absence of such factors was evident during recent pandemics, where poor surveillance infrastructure led to significant public health failures, even rendering airport screenings and contact tracing technology largely ineffective. These real-world events underline the urgency for strengthening these systems (13, 16).

**Figure 1:**
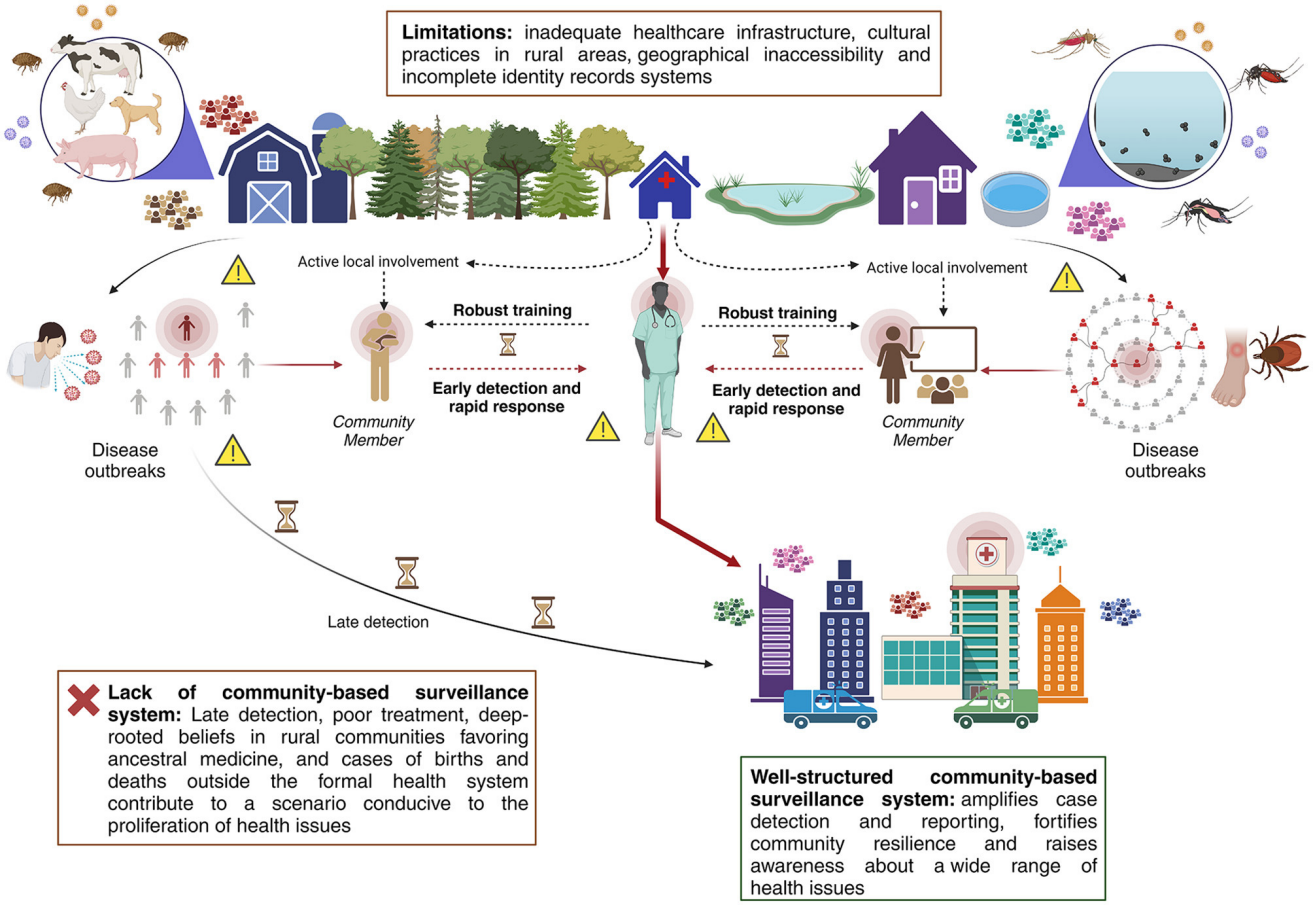
Graphic schematization of a community surveillance model along with its intimate link with the health system.

We recommend a balanced approach in the design and implementation of community-based surveillance systems, taking into consideration the unique operational environment and its impact on local communities and health systems. We firmly believe that the effectiveness of community-based surveillance systems significantly hinges on their interaction and interrelationship with the broader health system. Instead of considering them as external entities, it is essential to view them as integral extensions of the health system itself. Community-based surveillance not only expands the reach of the health system but also benefits from reciprocal support in the form of training, monitoring, evaluation, and continuous improvement provided by the health system to community surveillance actors (Figure 1). This synergy between community-based surveillance and the health system is vital for enhancing public health outcomes.

## Conclusion

In conclusion, a robust community-based surveillance system represents a multifunctional asset in public health. It enhances case detection and reporting, thereby strengthening the rapid response to health crises. Beyond this, it builds community resilience, fostering a proactive stance toward a spectrum of health concerns, ranging from infectious diseases to chronic non-communicable conditions. This comprehensive approach is particularly invaluable in resource-limited settings, where it addresses not only immediate health threats but also contributes to the long-term improvement of health outcomes. By integrating community-based surveillance into the wider health system, we can create a more resilient, responsive, and equitable public health framework, capable of meeting the diverse health needs of all communities.

## Author contributions

JI: Conceptualization, Data curation, Investigation, Methodology, Project administration, Resources, Supervision, Visualization, Writing – original draft, Writing – review & editing. AT-D-l-T: Investigation, Methodology, Validation, Visualization, Writing – original draft. PE: Investigation, Resources, Supervision, Validation, Writing – original draft. EO-P: Funding acquisition, Investigation, Methodology, Project administration, Supervision, Validation, Visualization, Writing – review & editing.
